# Oxidative Stress and Reprogramming of Mitochondrial Function and Dynamics as Targets to Modulate Cancer Cell Behavior and Chemoresistance

**DOI:** 10.1155/2019/4647807

**Published:** 2019-11-15

**Authors:** Stefano Falone, Michael P. Lisanti, Cinzia Domenicotti

**Affiliations:** ^1^Department of Life, Health and Environmental Sciences, University of L'Aquila, L'Aquila, Italy; ^2^University of Salford, Salford, UK; ^3^Department of Experimental Medicine, University of Genoa, Genoa, Italy

Among all the leading causes of mortality worldwide, cancer is responsible for approx. one in six deaths, and global cancer data show that the cancer burden has recently risen to 18.1 million people [1]. This represents a demanding challenge for healthcare systems and governments, whose actions are increasingly aimed at developing commitments for cancer treatment, on one hand, as well as at strengthening research activities on the mechanisms of carcinogenesis and cancer progression, on the other hand.

Fortunately, over the last decades, therapeutic strategies against cancer have taken significant steps forward, as demonstrated by the fact that age-standardized cancer death rates are falling globally [2]. However, medical treatments for oncological patients still encounter significant obstacles due to the development of radio- and chemoresistance, which, along with metastatic behavior, is thought to require extensive reprogramming of mitochondrial activity [3]. The balance of fission and fusion, along with the regulation of trafficking and autophagic removal, dictates mitochondrial morphology and function [4], and some researchers have suggested that mitochondrial dynamics could have a deep impact on redox homeostasis and antioxidant defense of cancer cells, as well as on their apoptotic response to oxidative stress-generating and DNA-damaging anticancer drugs [5].

This special issue comprises 4 review articles and 6 research articles that either investigated the role of mitochondria in mediating the proapoptotic response of malignant cells to anticancer drugs or examined ROS-dependent effects on redox-sensitive pathways controlling proliferation or viability of cancer cells.

Mitochondrial dynamics is in part regulated by the Liver Kinase B1- (LKB1-) AMP-activated protein kinase (AMPK) pathway [6]. LKB1 was identified as the critical upstream kinase required for AMPK activation thus providing a direct link between a known tumor suppressor and the regulation of metabolism. In fact, AMPK has a central role in the regulation of energy metabolism and coordinates glucose and lipid metabolism in response to alterations in nutrients and intracellular energy levels, contributing to maintain steady-state levels of intracellular ATP [7].

In their review article, F. Ciccarese et al. reported that loss of LKB1-AMPK signalling is able to confer sensitivity to energy depletion and to redox homeostasis impairment. Moreover, the authors have found an association between such a pathway and improved outcome in patients affected by advanced non-small-cell lung cancer (NSCLC) and treated with chemotherapy.

In this context, the review article of B. Poljsak et al. focused on the importance of understanding the origins of cancer in order to find successful strategies for effective cancer prevention and management.

In fact, it remains to be elucidated what exactly triggers the reprogrammed metabolism in cancer cells, and additional studies are needed to extend the knowledge about the relationships between metabolic abnormalities and the occurrence of genetic mutations in cancer.

As reviewed by B. Marengo et al., the metabolic reprogramming is the result of a complex network of mechanisms that, through the activation of oncogenes (i.e., MYC, HIF1, and PI3K) or the downregulation of tumor suppressors (i.e., TP53), induce an increased expression of glucose and/or glutamine transporters, along with an overexpression of glycolytic enzymes. The authors reported that among oncogenes, MYC is strongly involved in regulating cell metabolism since it facilitates glycolysis by inducing the activation of genes encoding for glycolytic enzymes and it is also able to promote mitochondrial biogenesis and function, thus increasing both oxygen consumption and ATP production.

In addition, it has been postulated that mitochondrial dysfunction in cancer cells would affect the cellular ATPase activities, ATP production, and subsequent apoptosis and migration processes [8].

In their research article, X. Zhang et al. demonstrated that the small molecule b-AP15 is an inhibitor of proteasome-associated deubiquitinase activity, which induced an increase in the generation of reactive oxygen species (ROS) in cancer cells. Oxidative stress (OS) induced by b-AP15 was found to be associated with a mitochondrial impairment and contributed to overcome resistance to bortezomib, which is an inhibitor of the 20S proteasome, in the clinical management of multiple myeloma.

Moreover, X. Li et al. have shown that KillerRed targeting mitochondria (mtKR) aggravated the mitochondrial dysfunction induced by radiation, thus suggesting a new strategy for ROS sensitization in future clinical cancer therapy. In this study, the N-terminal mitochondrial-targeting sequence (MTS) of PTEN-induced putative kinase 1 (Pink1) was used to mediate downstream mCherry and KillerRed to express in mitochondria, and the colocalization of mCherry (red) and mitochondrial tracker COX IV (green) was observed by fluorescence microscope analysis in COS-7 cells and human cervical cancer HeLa cells. In addition, the authors demonstrated in HeLa cells transfected with mtKR plasmids that mtKR induced mitochondrial ROS production, thus contributing to enhance apoptosis *via* the Cyt c/caspase-3 pathway in tumors treated with radiation.

Interestingly, evidence shows that natural molecules, such as curcumin and sulforaphane, are able to modulate the response of cancer cells to anticancer therapies. However, limited reports support the role of mitochondrial reprogramming in such a phenomenon, even though several natural chemosensitizers may act as regulators of mitochondrial dynamics and function. Further investigations on this may pave the way to diet-based approaches aimed at repressing the adaptive responses involving mitochondria following chemotherapy, thus contributing to an increase in the efficacy of anticancer strategies.

In their research article, B. George and H. Abrahamse, from University of Johannesburg, demonstrated that two phytochemicals isolated from roots of *Rubus fairholmianus* (1-(2-hydroxyphenyl)-4-methylpentan-1-one and 2-[(3-methylbutoxy) carbonyl] benzoic acid) were able to induce in human breast cancer MCF-7 cells an increase in ROS formation, cytochrome c release, and changes in mitochondrial membrane potential (MMP), thus activating the intrinsic apoptotic pathway. With specific focus on mitochondrion-dependent processes, the authors have quantitatively detected cytochrome c release by ELISA, as well as MMP by flow cytometry with a JC-1-based fluorescent kit.

The involvement of mitochondria in phytochemical-induced death response in cancer cells was even more evident in the original study of C. Antognelli et al., who identified in non-small-cell lung cancer (NSCLC) cells an interesting apoptogenic action of oleuropein (OP), a bioactive polyphenol found in olives. The authors found that OP was able to cause apoptotic death in A549 cells through depletion of mitochondrial superoxide anion, which in turn inhibited Akt signalling and activated the intrinsic apoptotic pathway *via* mitochondrial glyoxalase 2- (mGlo2-) mediated interaction with the proapoptotic protein Bax. This latter aspect is one of the most interesting findings of the work. In fact, the data provided by C. Antognelli et al. support the intriguing hypothesis that glyoxalase 2, an enzyme that is conventionally considered an enzyme committed to downregulate the formation of advanced glycation end products (AGEs) [9], is also able to form protein adducts with apoptosis-related factors. The critical role of mitochondrial redox reprogramming in the processes summarized above was demonstrated by silencing the mitochondrial superoxide dismutase (SOD2). This restored the normal O_2_^·-^ levels and mGlo2 expression, and in such conditions, OP failed to induce apoptosis in cancer cells. The interest for OP in terms of clinical application is increased since the authors demonstrated that OP did not affect the viability of cells derived from human normal bronchial epithelium.

The demand for anticancer drugs with low systemic adverse effects and low impact on healthy cells is highly appreciated. In this context, the work from Y. Zheng et al. (Guangzhou University, China) presented novel molecular targets of betulinic acid (BA), a pentacyclic triterpene derived from birch bark extracts. BA is attracting increasing attention due to its high selectivity for cancer cells, with no apparent systemic toxicity in mice [10, 11]. BA proapoptotic effects in malignant cells have been traditionally linked to mitochondrial ROS generation and induction of DNA damage [12, 13]; however, Y. Zheng et al. revealed that BA attenuated migration and invasion of highly aggressive breast cancer cells *via* aerobic glycolysis inhibition, and glucose-regulated protein (GRP78), a major chaperone in the endoplasmic reticulum, was found to be critical for inhibitory effects of BA on glycolytic proteins. Moreover, Y. Zheng et al.'s findings indicated that the oxygen consumption rate (OCR) of breast cancer cell lines MDA-MB-231 and BT-549 decreased following BA treatment, thus suggesting that BA switched the cells from an energetic metabolic state to a relatively quiescent state. In their experiments, the authors obtained accurate profiles of cancer cell energy phenotypes by using a live cell metabolic assay platform for extracellular flux analyses.

The crucial roles of ROS scavenging systems and mitochondria in triggering the cancer cell death induced by dietary polyphenols have been extensively reviewed and summarized by S. NavaneethaKrishnan et al. In their review, the authors focused their attention on some of the best known vegetable- and fruit-derived polyphenols with recognized pro-death properties against cancer cells. In particular, in their paper, S. NavaneethaKrishnan et al. provided interesting information about the cytotoxic effects of quercetin, curcumin, and resveratrol, with particular attention to the activation of ROS- and mitochondrion-dependent molecular pathways as possible mediators of such effects. In some cases, the redox-dependent cancer cell death is promoted through the activation of ROS-induced apoptosis, MMP reduction, cytochrome C release, and subsequent activation of caspase-3. In other cases, these polyphenols enhance TNF-related apoptosis-inducing ligand- (TRAIL-) induced apoptosis *via* the inhibition of ERK signalling pathway or by oxidatively modifying proteins that belong to the mitochondrial permeability transition pore (mPTP), thus causing mitochondrial depolarization, inhibition of ATP synthesis, and cell death. Furthermore, it was reported that some common plant-derived polyphenols exhibit a marked ROS-inducing capacity that leads to mitochondrial DNA damage and impairment of mitochondrial oxidative phosphorylation (OXPHOS). In addition, beyond exerting clear proapoptotic actions, some dietary polyphenols have also been proven to act as cell cycle arresting factors. Finally, the authors provided some interesting information about redox- and mitochondrion-targeting anticancer properties of less famous dietary polyphenols, such as capsaicin, coumaric acid, and phenethyl isothiocyanate, which leads to mitochondrial dysfunction in cancer cells but not in normal cells. Lastly, since poor absorption and fast metabolism of dietary polyphenols are concerning limiting factors in the administration to humans, promising strategies that include the use of novel formulations, prodrugs, and innovating delivery systems are proposed.

Hence, any strategy aimed at increasing ROS production or diminishing antioxidant capacity should be seen as a potential means by which the abnormal proliferation and growth of malignant cancer cells could be prevented or delayed. This topic was further investigated in the paper from K. Chen et al., who demonstrated that by deleting bloom syndrome protein (BLM), a DNA helicase belonging to the RecQ family, the proliferation of prostate cancer (PC) cells was repressed *via* downregulation of AKT signalling, and this was accompanied by enhanced ROS production. Of note, in their research, the authors used state-of-the-art techniques, such as isobaric tags for relative and absolute quantification (iTRAQ) proteomics, CRISPR/Cas9-mediated gene editing, and automated western blot quantitative analysis.

We sincerely hope that the articles offered by this special issue may provide interesting mechanistic insights of the role of mitochondria and redox-related signalling pathways in determining the cancer metabolic reprogramming, the proliferative activity of cancer cells, or their apoptotic response to exogenous stressors (e.g., natural anticancer molecules). We also strongly hope that further efforts will be spent for expanding the scientific knowledge on such topics, with the aim of future development of diet-based co-therapies for cancer.

Finally, we wish to thank all the authors for sharing their novel findings or reviews, and all reviewers for their priceless support in processing all the manuscripts.
